# The DnaA AAA+ Domain His136 Residue Directs DnaB Replicative Helicase to the Unwound Region of the Replication Origin, *oriC*

**DOI:** 10.3389/fmicb.2018.02017

**Published:** 2018-08-31

**Authors:** Yukari Sakiyama, Masahiro Nishimura, Chihiro Hayashi, Yusuke Akama, Shogo Ozaki, Tsutomu Katayama

**Affiliations:** Department of Molecular Biology, Graduate School of Pharmaceutical Sciences, Kyushu University, Fukuoka, Japan

**Keywords:** *E. coli*, *oriC*, DnaA, helicase, AAA+ family, protein–protein interaction

## Abstract

Chromosomal replication initiation requires dynamic mechanisms in higher-order nucleoprotein complexes that are constructed at the origin of replication. In *Escherichia coli*, DnaA molecules construct functional oligomers at the origin *oriC*, enabling localized unwinding of *oriC* and stable binding of DnaB helicases via multiple domain I molecules of *oriC*-bound DnaA. DnaA-bound DnaB helicases are then loaded onto the unwound region of *oriC* for construction of a pair of replisomes for bidirectional replication. However, mechanisms of DnaB loading to the unwound *oriC* remain largely elusive. In this study, we determined that His136 of DnaA domain III has an important role in loading of DnaB helicases onto the unwound *oriC*. DnaA H136A mutant protein was impaired in replication initiation *in vivo*, and in DnaB loading to the unwound *oriC in vitro*, whereas the protein fully sustained activities for *oriC* unwinding and DnaA domain I-dependent stable binding between DnaA and DnaB. Functional and structural analyses supported the idea that transient weak interactions between DnaB helicase and DnaA His136 within specific protomers of DnaA oligomers direct DnaB to a region in close proximity to single stranded DNA at unwound *oriC* bound to DnaA domain III of the DnaA oligomer. The aromatic moiety of His136 is basically conserved at corresponding residues of eubacterial DnaA orthologs, implying that the guidance function of DnaB is common to all eubacterial species.

## Introduction

**FIGURE 1:**
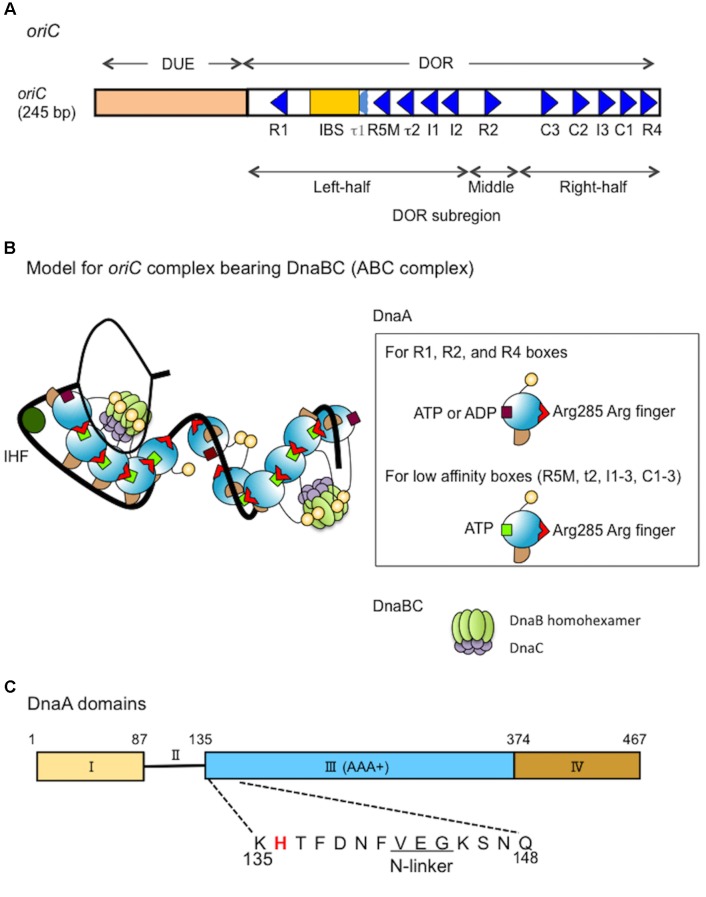
Structures of *oriC*, DnaA and the complex bearing DnaA and DnaBC. **(A)** Structure of *Escherichia coli oriC*. The duplex unwinding element (DUE) and DnaA-oligomerization region (DOR) are indicated. DnaA boxes (consensus sequence TTATNCACA) are shown by triangles, oriented to indicate sequence directionality (McGarry et al., 2004; Kawakami et al., 2005; Rozgaja et al., 2011; Shimizu et al., 2016). The R1 and R4 boxes have high affinity, the R2 box has moderate affinity and the others have low affinity for DnaA. A single integration host factor (IHF)-binding site (IBS) is present. When IHF binds to IBS, DnaA does not bind to the τ1 box (Sakiyama et al., 2017). **(B)** A possible structural model for *oriC*–DnaA complex carrying DnaBC complexes (ABC complex). DnaA domains are colored as in **C**. DnaA oligomers are constructed, and in one DnaA protomer, Arg285 “arginine finger” interacts with ATP bound to the flanking DnaA protomer (Kawakami et al., 2005; Erzberger et al., 2006). In the DnaA oligomers on *oriC* DOR (black line) (Rozgaja et al., 2011), Arg285 faces the middle of the DOR (Noguchi et al., 2015). DnaA domain IV can swivel using a short flexible loop present in its N-terminus, which supports DnaA oligomerization on DOR (Shimizu et al., 2016). In a crystal structure of the DNA-free oligomers of *A. aeolicus* DnaA domain III and domain IV, domain IV of one protomer interacts with domain III of the flanking protomer (Erzberger et al., 2002). By contrast, in molecular dynamics modeling of *E. coli* DnaA complexes at *oriC*, the structural changes induced by DNA binding and swiveling of domain IV prevents domain III-domain IV interaction (Shimizu et al., 2016). We propose that, near the time of initiation, when cellular ATP-DnaA levels peak (Kurokawa et al., 1999; Fujimitsu et al., 2009; Katayama et al., 2010), ATP-DnaA molecules bind to the low affinity DnaA box clusters R5M-τ2-I1-I2 and C3-C2-I3-C1 (Kawakami et al., 2005; Ozaki et al., 2008, 2012a; Keyamura et al., 2009; Sakiyama et al., 2017). We note that other studies (McGarry et al., 2004; Grimwade et al., 2007, 2018) using different methodologies to detect DnaA binding, report that R5M and C1 bind ATP-DnaA and ADP-DnaA with similar affinities. The illustration presents a model at the time of initiation. For stable DUE unwinding, ssDUE binds to DnaA domain III (Ozaki et al., 2008; Duderstadt et al., 2011; Sakiyama et al., 2017). Interactions between domain I of multiple DnaA protomers and each DnaB homohexamer promote stable binding (Abe et al., 2007; Keyamura et al., 2009). Deletion analysis of *oriC* supports the idea that each DnaA subcomplex (one on the left-half DOR and one on the right-half DOR) binds a single DnaB–DnaC complex (Ozaki and Katayama, 2012), although which DnaA protomer binds DnaB is unclear. In addition, the orientation of the DnaA subcomplex at the right-half DOR is important for efficient DnaB loading (Shimizu et al., 2016). When the DUE is unwound (thin black line), DnaB helicases are loaded onto the ssDNA regions and DnaC is released (Kaguni, 2011). For simplicity, DiaA is not shown in this model. **(C)** Domain structure of DnaA. His136 is highlighted in red and the N-linker motif is underlined. See text for details.

Chromosomal DNA replication is initiated by synergistic mechanisms involving multiple proteins with various functions. The initial steps of replication in *Escherichia coli* occur at the unique replication origin, *oriC*, which has a sophisticated structure that directs unwinding of duplex DNA and loading of replicative helicases (Kaguni, 2011; Leonard and Grimwade, 2015; Wolański et al., 2015; Katayama et al., 2017; **Figure 1A**). In these steps, the initiator DnaA molecules construct specific oligomers with the aid of DiaA (a DnaA-binding protein) and appropriately located DnaA-binding sequences (DnaA boxes) in the *oriC* DnaA-oligomerization region (DOR) (Leonard and Grimwade, 2015; Katayama et al., 2017). ATP-bound DnaA (ATP-DnaA), but not ADP-bound DnaA (ADP-DnaA), efficiently constructs homo-oligomers in a head-to-tail manner, with DnaA–DnaA interactions via the bound ATP and the Arg285 residue of flanking DnaA molecules (Kawakami et al., 2005; Erzberger et al., 2006; Ozaki et al., 2012a; Noguchi et al., 2015; Sakiyama et al., 2017; **Figures 1B,C**). The left-half DOR adjoins the *oriC* duplex unwinding element (DUE) and includes a specific binding site (IBS) for IHF (Integration host factor), a DNA-bending protein. The DnaA subcomplex including the left-half DOR and IHF causes localized unwinding of DNA in the DUE (Bramhill and Kornberg, 1988; Ozaki and Katayama, 2012; **Figure 1B**). The unwound single-stranded (ss) DUE is stabilized by binding to the DnaA oligomer, which is a prerequisite for DnaB loading (Ozaki et al., 2008; Ozaki and Katayama, 2012; Sakiyama et al., 2017).

DnaB helicase-loading includes critical processes for transition from replication initiation to DNA elongation. DnaB–DnaC complexes bind to DnaA oligomers that are bound to *oriC* (Keyamura et al., 2009; Kaguni, 2011; Ozaki and Katayama, 2012; Soultanas, 2012; Zawilak-Pawlik et al., 2017; **Figures 1B,C**). DnaB is a replicative helicase with a homohexamer structure and ring (or spiral) configuration (Kaguni, 2011; Itsathitphaisarn et al., 2012; Strycharska et al., 2013). DnaC acts in the loading of DnaB by stably binding to DnaB and promoting conformational changes in DnaB hexamer that are required for its loading on the ssDNA (Davey and O’Donnell, 2003; Galletto et al., 2003; Biswas and Biswas-Fiss, 2006; Makowska-Grzyska and Kaguni, 2010; Kaguni, 2011). DnaB C-terminal domain (CTD) is suggested to bind DnaC (Galletto et al., 2003). A pair of DnaB–DnaC complexes is thought to bind to an *oriC*–DnaA complex, resulting in a higher-order complex (Keyamura et al., 2009; Ozaki and Katayama, 2012; Ozaki et al., 2012a; Shimizu et al., 2016; **Figure 1B**): a deletion analysis of *oriC* suggests that each DnaA subcomplex constructed on the left- and right-half subregions of DOR binds a single DnaB helicase (or DnaBC complex) (Ozaki and Katayama, 2012). Also, deletion and insertion analyses suggest that the orientation of DnaA subcomplex constructed on the right-half DOR is optimized for efficient DnaB loading (Shimizu et al., 2016). The two DnaB helicases in the complex are loaded onto the ssDNA region of *oriC* in opposite directions each other, enabling bidirectional migration, leading to loading of one replisome on each strand (Fang et al., 1999; Carr and Kaguni, 2001; Ozaki and Katayama, 2012; Soultanas, 2012; Bell and Kaguni, 2013).

Unlike hyperthermophile bacterium *Aquifex aeolicus* DnaC (Mott et al., 2008), *E. coli* DnaC does not stably bind to DnaA (Keyamura et al., 2009). In *E. coli*, DnaB in DnaBC complex binds to DnaA complexes (Abe et al., 2007; Keyamura et al., 2009) and this mechanism is suggested to be conserved even in *A. aeolicus* (Mott et al., 2008). In addition, we recently demonstrate that YfdR, a protein encoded by an *E. coli* cryptic prophage, binds to DnaA depending on domain I Phe46 (a primary DnaB-binding site; also see below) and competes with the DnaBC complex in DnaA binding (Noguchi and Katayama, 2016). These results also support the notion that, in *E. coli*, DnaC *per se* does not directly bind DnaA, but does so indirectly as part of the DnaB–DnaC complex, in which DnaB binds to DnaA. However, dynamic mechanisms involved in DnaA–DnaB interactions have remained to be further elucidated.

The initiator protein DnaA has four functional domains (Kaguni, 2011; Katayama et al., 2017; **Figure 1C**). Domain I contains a specific site for binding to DnaB and DiaA (Sutton et al., 1998; Seitz et al., 2000; Abe et al., 2007; Keyamura et al., 2009). Domain II is a flexible linker between domains I and III (Nozaki and Ogawa, 2008). Domain III is the AAA+ domain that participates in nucleotide binding, ssDNA recruitment, and DnaA–DnaA interactions (Felczak and Kaguni, 2004; Iyer et al., 2004; Kawakami et al., 2005; Ozaki and Katayama, 2009; Duderstadt et al., 2011; Ozaki et al., 2012a,b). In addition, DnaA domain III N-terminal Val142–Gly144 constitutes a specific N-linker motif, which is thought to structurally interact with the adenine moiety of ATP/ADP (Smith et al., 2004), and our previous results demonstrate that the well-conserved Glu143 residue is specifically important for stable ATP/ADP binding (Ozaki et al., 2012b). DnaA domains II–III are also thought to have a weak binding site for DnaB (Marszalek et al., 1996; Seitz et al., 2000), although the precise location of this site has not been determined. DnaA domain IV binds directly to DnaA boxes, which has the 9-mer consensus sequence of (5′)TTATNCACA(3′) (Sutton and Kaguni, 1997; Fujikawa et al., 2003; Kaguni, 2011). In the N-terminus of domain IV, a short flexible loop enables the swiveling of this domain (Erzberger et al., 2002; Shimizu et al., 2016).

Functional mechanisms of DnaA–DnaB interaction for loading of DnaB on the ssDUE are thought to include multiple steps (Sutton et al., 1998; Seitz et al., 2000; Abe et al., 2007; Keyamura et al., 2009). DnaA domain I is the primary source of weak affinity for the DnaB CTD (Sutton et al., 1998; Seitz et al., 2000). We previously determined that a patch of DnaA that includes the Glu21 and Phe46 residues is exposed on the surface of domain I, binds to DnaB, and supports stable DnaB binding when DnaA oligomers are constructed on *oriC* (Abe et al., 2007; Keyamura et al., 2009). As DnaB is a homohexamer, binding of a single DnaB hexamer to multiple domain I molecules of a DnaA oligomer would effectively increase its affinity for DnaA, stabilizing DnaA–DnaB binding (Abe et al., 2007; Keyamura et al., 2009; Ozaki and Katayama, 2009; Zawilak-Pawlik et al., 2017). Although DnaA domain I is suggested to interact with a site including the N-terminus and its flanking region of DnaB CTD (Seitz et al., 2000), specific amino acids in the region have not been determined.

In addition to domain I, DnaA domains II–III are thought to contain a second site for DnaB binding, which is important for DnaB loading on *oriC* DNA (**Figure 1C**). A previous study assessed specific inhibition with monoclonal anti-DnaA antibodies, and the results suggested that the DnaA Pro111–Gln148 region includes a site for DnaB interaction (Marszalek and Kaguni, 1994; Marszalek et al., 1996; Sutton et al., 1998). Another study assessed functional interactions of various truncated forms of DnaA, and the results suggested that the DnaA Ser130–Gln148 region has a specific interaction site for the DnaB N-terminal domain (NTD) (Seitz et al., 2000). Deletion analysis of domain II has demonstrated that the DnaA Ala99–Val134 region is largely dispensable for DnaA functions in replication initiation at *oriC* (Nozaki and Ogawa, 2008). Taken together, these results suggest that the DnaA domain III N-terminus spanning Lys135–Gln148 might contain the second essential site for DnaB interaction (**Figure 1C**).

In this study, to determine the role for the second DnaB-binding site of DnaA, we extended functional analysis of the DnaA domain III N-terminus to the region spanning Lys135–Gln148. Alanine-scanning experiments revealed that His136, Phe141, and Val142 are crucial for complementation of *dnaA46* temperature-sensitive mutations. Our previous study indicated that Val142 is an essential constituent of the N-linker (Ozaki et al., 2012b), and Phe141 is its flanking bulky residue. Thus, in this study, we focused our attention on analyses on His136 and found that a substitution of this residue (H136A) impaired specifically DnaA-dependent loading of DnaB on ssDUE, without affecting its assembly at *oriC*, unwinding of DUE, or recruitment of ssDUE. A structural model for the *oriC*–DnaA complex is compatible with a predicted role for His136 in directing DnaB for loading on the ssDUE.

## Materials and Methods

### Nucleic Acids

Plasmid pKA234, a derivative of the pING vector which has an arabinose-inducible promoter, was used for overproduction of wild-type DnaA, and has been described previously (Ozaki et al., 2012b). Derivatives of pKA234 encoding DnaA variants with individual alanine substitutions for each residue from Lys135A to Gln148 were constructed with specific mutagenic primers and QuikChange site-directed mutagenesis protocol (Stratagene [Agilent], Agilent, La Jolla, CA, United States), as previously described (Ozaki et al., 2012b).

The DOR dsDNA fragment (ΔDUE) and 28-mer T-rich ssDUE strand have been described previously (Ozaki and Katayama, 2012). M13KEW101 and pBSoriC are *oriC* plasmids containing intact *oriC*, and pBSoriCΔR4-R2 is a derivative of pBSoriC with only the left half of *oriC*. M13KEW101, pBSoriC, and pBSoriCΔR4-R2 have been described previously (Kawakami et al., 2005; Ozaki and Katayama, 2012). pBSoriCΔDUE is a DUE-deleted derivative of pBSoriC, constructed by outward-directed PCR with pBSoriC as a template and primers ori-1 and MR28r1-r, as described previously (Ozaki et al., 2008; Ozaki and Katayama, 2012); the amplified DNA was digested with *Hinc*II and self-ligated, resulting in pBSoriCΔDUE. Biotinylated *oriC* DNA (bio-*oriC*) has been described previously (Keyamura et al., 2009). M13-A-site ssDNA is a derivative of M13 ssDNA with a hairpin structure that contains a DnaA-box sequence (Masai et al., 1990).

### Buffers

Buffer G contained 20 mM HEPES-KOH (pH 7.6), 5 mM magnesium acetate, 1 mM EDTA, 10% glycerol, 0.1% Triton X-100, and 4 mM dithiothreitol (DTT). Buffer F contained 20 mM Tris–HCl (pH 7.5), 8 mM DTT, 10 mM magnesium acetate, 125 mM potassium glutamate, 3 mM ATP, and 0.5 mg/mL bovine serum albumin (BSA). Buffer ABC contained 20 mM Tris–HCl (pH 7.5), 0.1 mg/mL BSA, 8 mM DTT, 8 mM magnesium acetate, 0.01% Brij-58, and 125 mM potassium glutamate.

### DnaA Proteins

Wild-type DnaA and DnaA H136A proteins were overproduced in *E. coli* strain KA450 [Δ*oriC1071*::Tn*10 rnhA199*(Am) *dnaA17*(Am)] from pKA234 or pH136A (a derivative of pKA234 encoding DnaA H136A), and purified as previously described (Ozaki et al., 2012b).

### Flow-Cytometry Analysis

Flow cytometry was performed as described previously (Noguchi et al., 2015; Inoue et al., 2016; Sakiyama et al., 2017). Briefly, cells were grown in LB medium at 30°C to an absorbance at 660 nm of 0.2, then incubated at 42°C for 150 min. Before and after 42°C incubation, aliquots were withdrawn for analysis of cell mass (or cell volume) with a FACSCalibur flow cytometer (BD Biosciences, Franklin Lakes, NJ, United States). The remaining aliquots of the cell cultures were further incubated in the presence of rifampicin and cephalexin for 4 h, followed by analysis of DNA content by flow cytometry.

### *oriC* Plasmid Replication Assay

The assay was performed essentially as described previously (Keyamura et al., 2009). Briefly, a crude protein extract containing proteins required for *oriC* replication (except for DnaA) was prepared from *E. coli* strain WM433 (*dnaA204*) (Fuller et al., 1981). Reactions (25 μL) were performed with purified DnaA, M13KEW101 *oriC* plasmid (38 fmol as plasmid; 600 pmol nucleotides), and WM433 crude extract (200 μg), as described (Keyamura et al., 2009).

### P1 Nuclease Assay for DUE Unwinding

The assay was performed essentially as described previously (Ozaki et al., 2012b). Briefly, ATP-DnaA or ADP-DnaA was incubated for 3 min at 38°C with M13KEW101 *oriC* plasmid (12 fmol as plasmid; 400 ng) and HU protein (16 ng), followed by brief incubation with P1 nuclease. DNA was purified and incubated with restriction enzyme *Bse*RI, followed by analysis by agarose-gel electrophoresis, with ethidium bromide staining.

### ssDUE-Recruitment Electrophoretic Mobility Shift Assay (EMSA)

The assay was performed as described previously (Ozaki and Katayama, 2012; Ozaki et al., 2012a; Sakiyama et al., 2017). Briefly, DnaA and the DUE-deleted *oriC* dsDNA fragment, DOR(ΔDUE) (5 nM) were incubated on ice for 5 min, followed by incubation for 5 min at 30°C with ^32^P-labeled 28-mer T-rich ssDUE strand (2.5 nM) in the presence of λphage DNA (25 ng). The resultant DNA complexes were analyzed by 4% polyacrylamide-gel electrophoresis at 4°C.

### Form I^∗^ Assay

The assay was performed as previously described (Ozaki and Katayama, 2012; Noguchi et al., 2015; Shimizu et al., 2016). Briefly, ATP-DnaA and pBSoriC or pBSoriCΔR4-R2 (1.6 nM) were incubated for 15 min at 30°C in buffer (25 μL) containing 760 nM SSB, 76 nM GryA, 100 nM His-GyrB, 42 nM IHF, 100 nM His-DnaB, and 100 nM His-DnaC. Reactions were terminated by addition of 1% SDS, and the DNA samples were purified and analyzed by 0.65% agarose-gel electrophoresis and ethidium bromide staining.

### Biotin-Tagged *oriC* Pull-Down Assay

The assay was performed as previously described (Keyamura et al., 2009; Ozaki and Katayama, 2012; Ozaki et al., 2012a; Noguchi and Katayama, 2016). Briefly, bio-*oriC* (419 bp, 100 fmol), including the entire *oriC* and flanking regions (Keyamura et al., 2007), was incubated on ice for 10 min in buffer G (10 μL) containing DnaA and 1 mM ATP. The bio-*oriC* and bound proteins were recovered by pull-down with streptavidin-coated beads (Promega, Madison, WI, United States), washed twice with buffer G (12.5 μL) containing 75 mM KCl, and dissolved in SDS sample buffer. Proteins were quantified using silver staining and quantitative protein standards, as we previously performed (Ozaki and Katayama, 2012; Noguchi and Katayama, 2016). The recovered amounts of bio-*oriC* were deduced by quantifying bio-*oriC* remaining in supernatants. For analysis of DnaB binding to *oriC*–DnaA complexes, bio-*oriC* was coincubated with DnaA and DnaB in the presence of absence of DnaC, followed by the wash step conducted only once with buffer G excluding KCl.

### His-DnaB Pull-Down Assay

The assay was performed under similar conditions to the Form I^∗^ assay. ATP-DnaA (90 nM) and pBSoriC or pBSoriCΔDUE (1.6 nM) were incubated for 15 min at 30°C in buffer F (10 μL) containing 40 nM IHF, 200 nM His-DnaB K236A, and the native (non-tagged) DnaC (200 nM). After addition of Co^2+^-conjugated magnetic beads (1 μL bed volume: Dynabeads, Invitrogen, Carlsbad, CA, United States) and incubation for 15 min at 4°C, the beads and bound materials were collected by magnetic pull-down and washed in buffer F containing 100 mM NaCl and excluding BSA. His-DnaB-bound plasmid DNA was eluted in standard SDS sample buffer, and analyzed by 1% agarose-gel electrophoresis and ethidium bromide staining.

### ABC Primosome Assay

The assay was performed as described previously (Abe et al., 2007; Keyamura et al., 2009). Briefly, the indicated amounts of DnaA were incubated at 30°C for 15 min in buffer ABC (25 μL) containing M13-A-site ssDNA (1.1 nM as ssDNA; 220 pmol nucleotides), 0.5 μg SSB, 65 ng DnaB, 65 ng DnaC, 72 ng DnaG, 108 ng DNA polymerase III^∗^, 26 ng β-clamp subunit, 1 mM ATP, 0.25 mM each of GTP, CTP, and UTP, and 0.1 mM each of dNTP and [α-^32^P]dATP. DNA polymerase III^∗^ is a subcomplex of DNA polymerase III holoenzyme lacking the β-clamp subunit. Reactions were stopped by addition of 1 mL 10% trichloroacetic acid, and the amounts of synthesized DNA were measured by liquid scintillation.

## Results

### DnaA His136 Is Essential for Initiation Activity *in vivo*

A putative DnaB interaction site has been speculated to reside in a DnaA domain III N-terminal region spanning Lys135–Gln148. We conducted an alanine-scanning analysis on all the amino acid residues in this region except for Gly144, which lacks a side chain. Plasmid pKA234 contains the wild-type *dnaA*-coding region downstream of the arabinose-inducible P_BAD_ promoter (Kubota et al., 1997). Derivatives of pKA234 containing *dnaA* mutations encoding alanine substitutions were constructed and used for complementation tests with a temperature (42°C)-sensitive *dnaA46* host strain (KA413). Even in the absence of the inducer arabinose, introduction of pKA234, but not the vector pING1, enabled the KA413 cells to grow at 42°C, presumably because of leaky expression of the pKA234 *dnaA* gene (**Table 1**). In similar experiments that we previously performed, DnaA amounts in cells bearing pKA234 was 1.3- to 2.8-fold higher than those in cells bearing pING1 (Kawakami et al., 2005; Ozaki et al., 2008). Unlike pKA234, plasmids containing *dnaA* alleles encoding H136A, F141A, or V142A substitutions did not complement the *dnaA46* temperature (42°C) sensitive growth (**Table 1**).

**Table 1:** Results of plasmid complementation.

		Transformation efficiency (cfu/μg DNA)		Ratio (42/30°C)
			
Plasmid	*dnaA* allele	30°C	42°C	
pING1(vector)	None	2.8 × 10^6^	<1.1 × 10^3^	<3.9 × 10^-4^
pKA234	Wild-type	2.0 × 10^5^	3.1 × 10^5^	1.6
pK135A	*K135A*	2.1 × 10^6^	1.5 × 10^6^	1.4
pH136A	*H136A*	1.3 × 10^6^	<1.1 × 10^3^	<8.5 × 10^-4^
pT137A	*T137A*	1.4 × 10^6^	8.0 × 10^5^	0.57
pF138A	*F138A*	1.5 × 10^6^	9.0 × 10^5^	0.60
pD139A	*D139A*	1.3 × 10^6^	6.6 × 10^5^	0.51
pN140A	*N140A*	1.5 × 10^6^	9.0 × 10^5^	1.6
pF141A	*F141A*	1.5 × 10^6^	<2.0 × 10^3^	<1.3 × 10^-3^
pV142A	*V142A*	5.6 × 10^6∗^	<1.1 × 10^3^	<2.0 × 10^-4^
pE143A	*E143A*	9.6 × 10^5^	2.1 × 10^6^	2.2
pK145A	*K145A*	1.9 × 10^6^	1.2 × 10^6^	1.6
pS146A	*S146A*	1.5 × 10^6^	1.5 × 10^6^	1.0
pN147A	*N147A*	6.5 × 10^6∗^	8.9 × 10^6^	1.4
pQ148A	*Q148A*	2.0 × 10^6^	1.3 × 10^6^	1.5


*Plasmids were introduced into *Escherichia coli* KA413 (*dnaA46*) cells, which were incubated either at 30°C for 24 h or at 42°C for 12 h on LB-agar plates containing 50 μg/mL thymine and 100 μg/mL ampicillin. The colonies were then counted as described previously (Nishida et al., 2002; Ozaki et al., 2012a). Transformation efficiency [colony forming units (cfu)/μg DNA] at each temperature, and ratios of efficiencies, are shown. ^∗^Only tiny colonies were observed by 24 h, so incubation was prolonged to 36 h before colony counting.*

Val142 is a component of the N-linker motif (Val142-Glu143-Gly144), a conserved sequence in AAA+ family proteins that is thought to interact with the adenine moiety of ATP (Smith et al., 2004). DnaA V142A has previously been shown to cause overinitiation of replication at 30°C in the absence of wild-type DnaA presumably because its binding to ADP is unstable, resulting in rapid exchange of bound ADP to ATP (Ozaki et al., 2012b), and here consistently, we observed that DnaA V142A resulted in slow colony formation at 30°C and severe inhibition of colony formation at 42°C (**Table 1**). DnaA F141A also resulted in growth inhibition at 42°C, suggesting that substitution of the hydrophobic aromatic side chain of phenylalanine resulted in DnaA structural changes that indirectly inhibited the function of the N-linker motif residues, such as Val142.

We previously indicated that Glu143 within the N-linker motif is important for stable binding of ATP and ADP, and DnaA E143A causes moderate overinitiation of replication at 30°C in the absence of wild-type DnaA, presumably because of rapid exchange of bound ADP to ATP (Ozaki et al., 2012b). When co-expressed with the wild-type DnaA, DnaA E143A was shown not to cause inhibition of cell growth (Ozaki et al., 2012b), which might be a consequence of formation of mixed complexes on *oriC* repressing overinitiation (Ozaki et al., 2012b). These previous observations are consistent with the current data of DnaA E143A at 30°C (**Table 1**). When cells were incubated at 42°C, a temperature at which replication initiation is stimulated even in wild-type cells, a severe inhibitory effect of DnaA E143A for cell growth by overinitiation might be suppressed. Based on these ideas, we excluded DnaA F141A and V142A for further analyses in this study.

**FIGURE 2:**
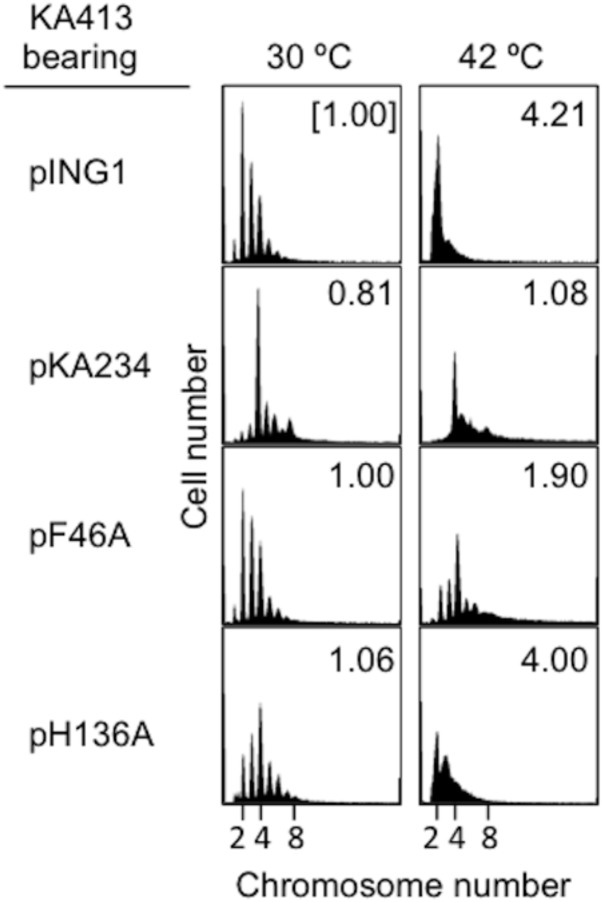
Flow-cytometry analysis. KA413 (*dnaA46*) cells containing the indicated plasmids were grown to exponential phase in LB medium containing 50 μg/mL thymine and 50 μg/mL ampicillin at 30°C, and further incubated at 42°C for 150 min. Before and after 42°C incubation, portions of the cultures were withdrawn for analyses of cell size (mass) and DNA content by flow cytometry. Chromosome numbers per cell corresponding to each peak are indicated. Mean cell mass relative to that of KA413 cells containing pING1 and grown at 30°C is indicated at the top right corner of each panel. pKA234 is a DnaA-expressing derivative of pING1. pF46A and pH136A are derivatives of pKA234 encoding variants of DnaA with single amino acid substitutions.

To further elucidate initiation activity *in vivo* of DnaA H136A, we used flow cytometry to determine the chromosomal replication modes of *dnaA46* cells containing derivatives of pKA234. Cells were cultured for exponential growth at 30°C and shifted to 42°C for inactivation of the intrinsic DnaA46. Further incubation with rifampicin and cefalexin enabled run-out replication of the chromosomes. At 30°C, distinct DNA peaks were seen for each strain (**Figure 2**). In *dnaA46* cells with the pING vector, the two-chromosome peak predominated, with minor peaks for one, three, four, and five chromosomes, indicating asynchronous initiations (Skarstad et al., 1986, 1988). Introduction of wild-type *dnaA* (pKA234) into *dnaA46* cells stimulated initiation and resulted in a predominant four-chromosome peak. DnaA F46A protein is impaired in stable DnaB binding and DnaB loading at *oriC* (Abe et al., 2007; Keyamura et al., 2009). Introduction of the *dnaA F46A* allele as a negative control (pF46A) gave a similar profile to that of *dnaA46* cells with the pING vector, whereas moderate stimulation was detected with *dnaA H136A* (pH136A). This stimulation could be a consequence of mixture of DnaA46 and DnaA H136A proteins (see below). At 42°C, the profiles with pING and *dnaA H136A* were fundamentally similar with respect to inhibition of initiation, whereas moderate stimulation occurred with *dnaA F46A*, as previously observed (Abe et al., 2007; Keyamura et al., 2009). These results suggest that DnaA H136A is impaired in the initiation of chromosomal replication *in vivo*. In addition, the smeared peaks of *dnaA H136A* culture at 42°C suggest that progression of replication forks also are moderately inhibited by this mutation. Abnormal interaction between DnaA H136A and DnaB helicases might inhibit fork progression in addition to replication initiation *in vivo*.

### Purified DnaA H136A Protein Sustains ATP Binding, but Is Inactive in Initiation *in vitro*

**FIGURE 3:**
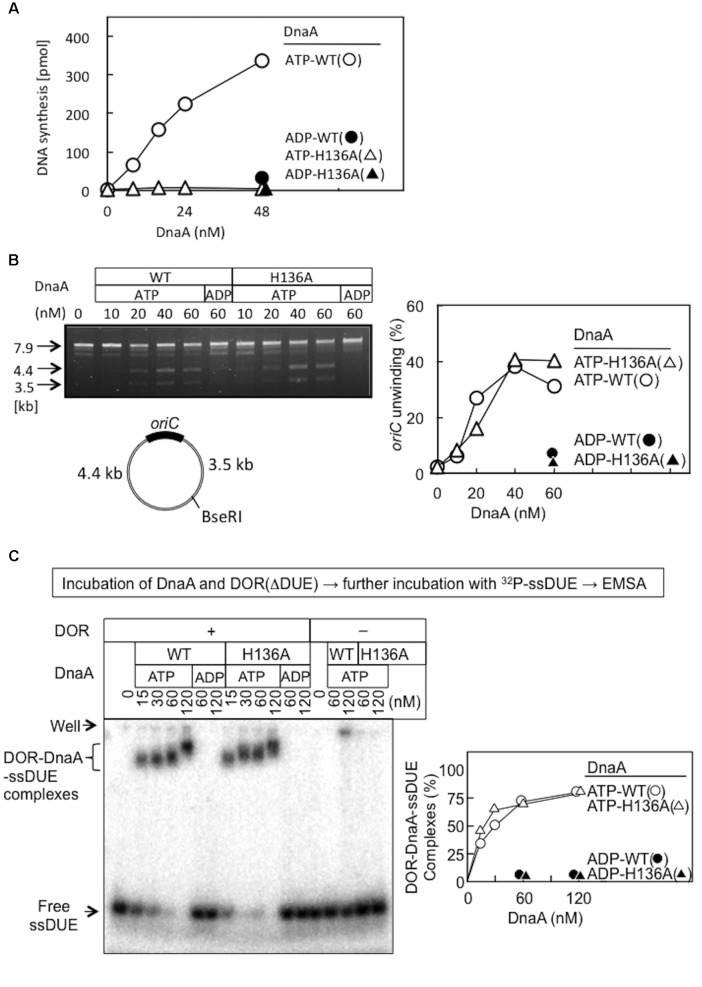
Activities of wild-type (WT) and H136A mutant DnaA in replication initiation and *oriC* unwinding. **(A)** *In vitro oriC* replication assay. Nucleotide-bound WT and H136A DnaA were incubated at 30°C for 20 min with a replicative WM433 (*dnaA204*) crude extract, M13KEW101 *oriC* plasmid (600 pmol nucleotides), and dNTPs containing [α -^32^P]dATP, as described previously (Nishida et al., 2002). Similar results were obtained in an independent experiment. **(B)** P1-nuclease assay. Nucleotide-bound forms of DnaA were incubated with M13KEW101 *oriC* plasmid and HU protein, followed by incubation with P1 nuclease and then with *Bse*RI. The *Bse*RI restriction site is located distally to *oriC*, so that two DNA fragments (3.5 and 4.4 kb) are produced if *oriC* is unwound and digested by P1 nuclease. Digested samples were analyzed by 1% agarose-gel electrophoresis. *oriC* unwinding (3.5 and 4.4 kb fragments) was quantified. **(C)** Nucleotide-bound forms of DnaA and an *oriC* DOR(ΔDUE) fragment (5 nM) were incubated on ice for 5 min, then for 5 min at 30°C with ^32^P-labeled 28-mer ssDUE (2.5 nM). Formation of protein–DNA complexes was analyzed by 4% polyacrylamide-gel electrophoresis, and quantified.

DnaA H136A protein was overproduced and purified, and shown to have high-affinity binding of ATP and ADP at levels similar to wild-type DnaA (**Table 2**), suggesting preservation of the overall protein structure of domain III. By contrast, when replication initiation was assessed with an *oriC* plasmid and a replicative-protein extract, even the ATP form of DnaA H136A was inactive, unlike that of wild-type DnaA (**Figure 3A**).

**Table 2:** Binding of ATP and ADP by wild-type (WT) and H136A DnaA.

	*K_D_* (nM)		Stoichiometry	
		
DnaA	ATP	ADP	ATP	ADP
WT	24	35	0.24	0.17
H136A	21	50	0.10	0.13


*DnaA protein (76 nM) was incubated on ice for 15 min with various concentrations of [α-^*32*^P]ATP and [2,8-^*3*^H]ADP. Nucleotide-bound DnaA was captured on nitrocellulose filters, levels of bound nucleotides were determined by liquid-scintillation counting, and affinities of DnaA proteins for ATP or ADP were deduced from Scatchard plots, as previously described (Ozaki et al., 2012a,b).*

### DnaA H136A Is Active in *oriC* Unwinding and ssDUE Binding

Functions of DnaA His136 were assessed with reconstituted systems for *oriC* unwinding and ssDUE binding. In the P1-nuclease assay, unwinding of *oriC* DUE by initiation complexes produces ssDNA that is sensitive to endonuclease P1. In this assay, the ATP forms of wild-type DnaA and DnaA H136A demonstrated similar activities in specific *oriC* unwinding (**Figure 3B**).

To determine the abilities of these proteins to stabilize the unwound DUE, we analyzed ssDUE-binding activity by electrophoretic mobility shift assay (EMSA) using ssDUE, *oriC* DOR, and DnaA. In this assay, ATP-DnaA molecules can construct homo-oligomers on the DOR and bind ssDUE with high affinity, producing DOR-DnaA-ssDUE ternary complexes (Ozaki et al., 2008; Ozaki and Katayama, 2012; Noguchi et al., 2015; Sakiyama et al., 2017). Construction of the ternary complexes has been shown to occur with ATP-DnaA (but not ADP-DnaA) and to require specific DnaA residues involved in construction of *oriC* open complexes (including AAA+ arginine-finger Arg285 and ssDUE-binding H/B motifs Val211 and Arg245) (Ozaki et al., 2008; Ozaki and Katayama, 2012). Here, the ATP form of DnaA H136A resulted in binding of the ssDUE to the DnaA–DOR complexes at a level similar to that seen with wild-type DnaA (**Figure 3C**). DnaA-ssDUE binding was dependent on DOR as we previously demonstrated. Faint signals in the gel wells were irregular aggregates of DnaA which involved ^32^P-ssDUE. Those were unstable and slowly degraded during electrophoresis, resulting in faint smeared bands (Ozaki et al., 2008; Ozaki and Katayama, 2012; Sakiyama et al., 2017). These results indicate that DnaA H136A is fully active in the primary reactions required for DUE unwinding and stable binding of ssDUE, which are prerequisites for DnaB loading on ssDUE.

### DnaA H136A Is Impaired in DnaB Loading Onto Unwound *oriC*

We conducted the Form I^∗^ assay to determine DnaB loading onto the unwound strands of *oriC* (via indirect interactions with DnaC and DnaA) and its subsequent helicase action on DNA strands. Loading on ssDUE activates DnaB helicase, expanding the ssDNA region and introducing positive supercoils. Activation of gyrase then produces highly negatively supercoiled *oriC* plasmid (Form I^∗^), and this topoisomeric form is distinguished from Form I by gel electrophoresis (Baker et al., 1986).

**FIGURE 4:**
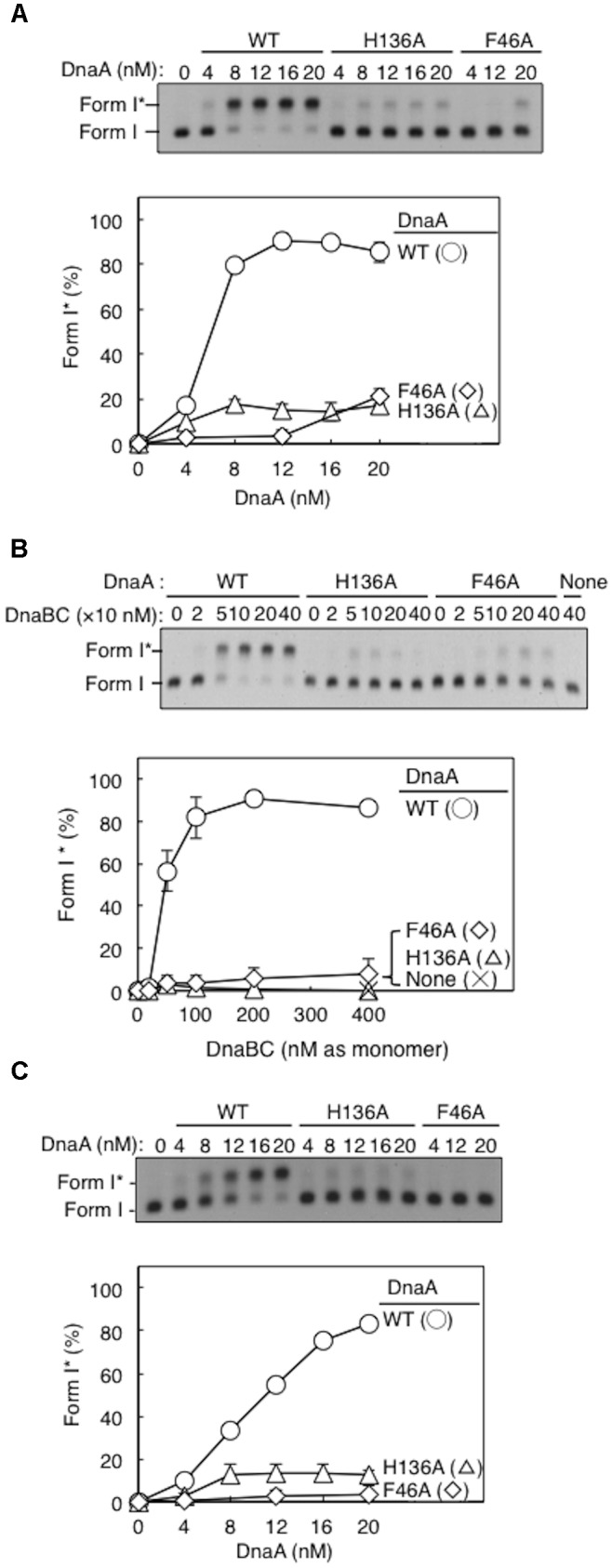
DnaA-directed loading of DnaB at *oriC*. Representative gel images are shown in black–white inverted mode, and migration positions of negatively supercoiled (Form I) and highly negatively supercoiled (Form I^∗^) plasmid DNA are indicated. Form I^∗^ is produced from Form I by DnaB helicase and DNA gyrase activities. Relative amounts of Form I^∗^ to input DNA were quantified as “Form I^∗^ (%),” and mean values with SD (*n* = 2) are shown in each graph. WT, wild-type DnaA; F46A, DnaA F46A; H136A, DnaA H136A; None, no DnaA. **(A)** Form I^∗^ assay with WT, F46A, and H136A DnaA. Indicated amounts of ATP-DnaA were incubated for 15 min at 30°C in buffer containing DnaB–DnaC (100 nM), SSB, gyrase, IHF, and pBSoriC *oriC* plasmid Form I. The resultant DNA forms were analyzed by agarose-gel electrophoresis. **(B)** Form I^∗^ assay with fixed concentration (20 nM) DnaA and various concentrations DnaB–DnaC complexes. Other conditions were as in **(A)**. **(C)** Form I^∗^ assay, as in **(A)**, but with pBSoriCΔR4-R2 plasmid.

Form I^∗^ production was severely impaired for DnaA H136A, even at high levels (**Figure 4A**). Compared with wild-type DnaA, DnaA H136A, and the negative control DnaA F46A (which is inactive in primary DnaB binding) only had low levels of Form I^∗^ production, even in the presence of excessive amounts of DnaBC proteins (**Figure 4B**). The left-half *oriC* is a minimal region for DUE unwinding and DnaB loading (Ozaki and Katayama, 2012; Sakiyama et al., 2017). Compared with wild-type DnaA, DnaA H136A, and DnaA F46A also produced only low levels of Form I^∗^ even with left-half *oriC* (**Figure 4C**), consistent with the idea that DnaB loading reactions *per se* are inhibited also with the full-length *oriC*, but not with the idea that DnaBC complexes binds simultaneously to left- and right-half DnaA–*oriC* subcomplexes, causing abortive interactions with each other, and inhibiting DnaB loading on ssDUE. Taken together, the results suggest that the DnaA His136 residue has a predominant role in the process of productive loading of DnaB to unwound *oriC*.

### A Subgroup of DnaA Molecules in an *oriC* Complex Requires His136 for DnaB Loading

**FIGURE 5:**
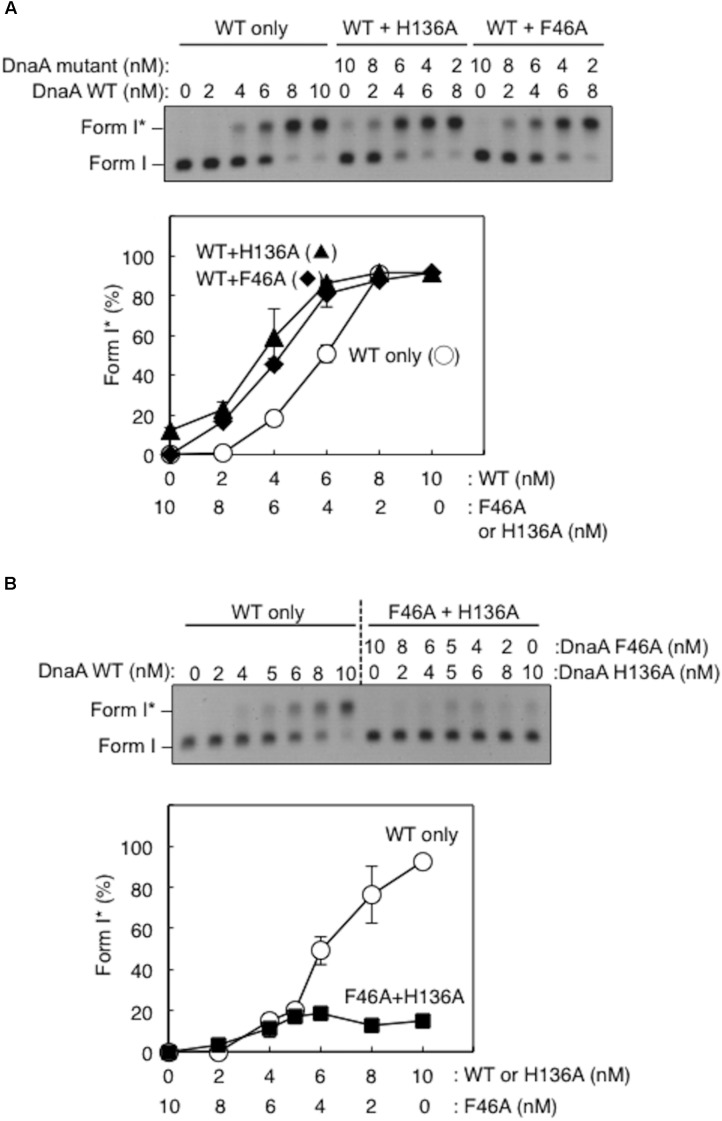
Activities of DnaB loading by mixtures of wild-type (WT) and mutant DnaA. Form I^∗^ assays were performed with different combinations of WT, F46A, and H136A DnaA, with pBSoriC *oriC* plasmid. Form I^∗^ is highly negatively supercoiled, and is produced from Form I by DnaB helicase and DNA gyrase activities. Reactions were incubated for 15 min at 30°C. Representative gel images are shown in black–white inverted mode. Amounts of Form I^∗^ relative to the input DNA were quantified as “Form I^∗^ (%),” and mean values with SD (*n* = 2) are shown. **(A)** Mixtures of WT DnaA and DnaA F46A or H136A were used. **(B)** Mixtures of DnaA F46A and DnaA H136A were used.

Here, activity of mixtures of the wild-type DnaA and DnaA H136A or DnaA F46A proteins were analyzed using Form I^∗^ assay. In these experiments, complexes including both the wild-type DnaA and H136A (or F46A) proteins should be constructed on the same *oriC* molecule. Thus, if a subgroup of DnaA protomers is not used for DnaB loading, the partial inclusion of DnaA H136A (or F46A) which is fully active in DUE unwinding, might retain DUE unwinding and DnaB loading. DnaA F46A, although inactive for DnaB binding by itself, can help achieve optimal Form I^∗^ formation when wild-type DnaA is provided suboptimally (**Figure 5A**), as previously demonstrated (Keyamura et al., 2009), suggesting that DnaA F46A can contribute to DnaB loading when it is a part of a complex including the wild-type DnaA at *oriC*. This means that only a subgroup (but not all) of DnaA molecules assembled on *oriC* would require Phe46 for DnaB loading (Keyamura et al., 2009). Here, we obtained similar results with DnaA H136A in the presence of wild-type DnaA (**Figure 5A**).

DnaA H136A and DnaA F46A proteins were mixed at various ratios and assessed by Form I^∗^ assay, but did not produce substantial amounts of Form I^∗^ (**Figure 5B**). Only weak Form I^∗^ formation due to the residual activity of DnaA H136A was observed. These results suggest that, at least in a subgroup of DnaA molecules assembled on *oriC*, both the His136 and Phe46 residues must be present in the same DnaA protomer for DnaB loading.

### DnaA H136A Forms Stable *oriC*–DnaA Complexes, but Results in Impaired Loading of DnaB on Unwound *oriC*

**FIGURE 6:**
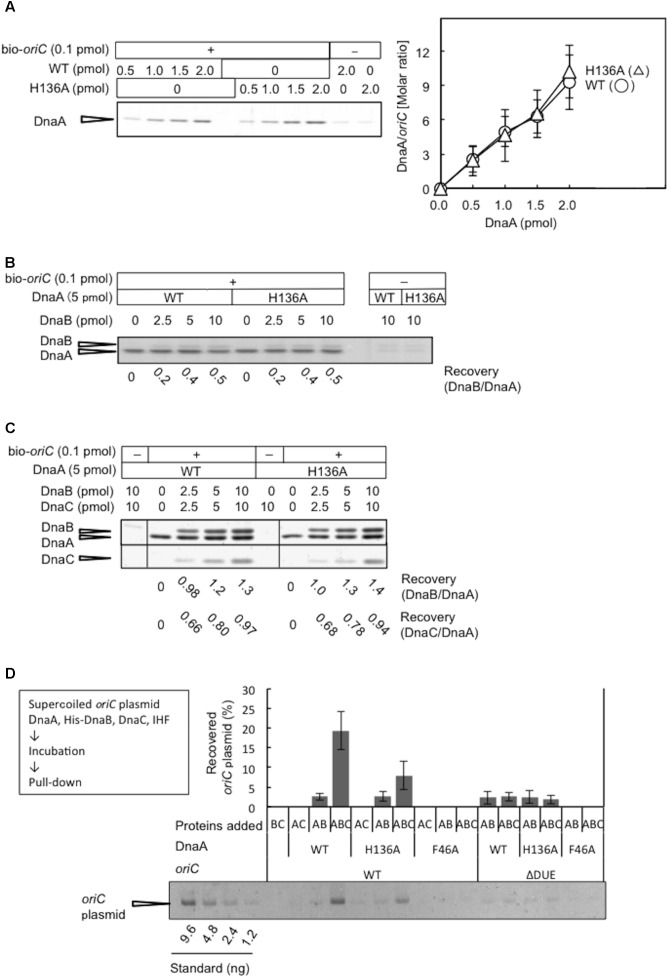
DnaA assembly on *oriC* and DnaB loading on unwound *oriC*. **(A)** Biotinylated *oriC* DNA (bio-*oriC*) pull-down assay with wild-type (WT) and H136A mutant DnaA. The bio-*oriC* (0.1 pmol) was incubated on ice for 10 min with indicated amounts of ATP-DnaA, and bound materials were recovered with streptavidin-coated beads. Recovered DnaA was analyzed by SDS-11% polyacrylamide gel electrophoresis, with silver staining (left panel) and the number of DnaA molecules bound to *oriC* was deduced using quantitative standards (right panel). Experiments were performed in triplicate, and a representative gel image and the means with SD are shown. **(B)** bio-*oriC* pull-down of DnaA and DnaB proteins. Similar to the above, bio-*oriC* was incubated with 5 pmol ATP-DnaA (WT or H136A) and different amounts of DnaB, and bound materials were recovered with streptavidin-coated beads. Ratios of bound DnaB (as monomers) and DnaA were calculated using quantitative standards. **(C)** bio-*oriC* pull-down of DnaA, DnaB, and DnaC proteins. Similar experiments were performed in the presence of DnaC. Ratios of bound DnaC (as monomers) and DnaA as well as bound DnaB (as monomers) and DnaA were calculated, using quantitative standards. **(D)** His-DnaB pull-down assay. ATP-DnaA (90 nM, WT, H136A, or F46A) was incubated for 15 min at 30°C in Form I^∗^ buffer containing pBSoriC or pBSoriCΔDUE (1.6 nM), IHF (40 nM), and His-DnaB (200 nM), with or without non-tagged DnaC (200 nM). His-DnaB-bound *oriC* plasmids were collected with magnetic beads, and analyzed by 1% agarose-gel electrophoresis. The percentages of recovered DNA relative to the input DNA were quantified and indicated as “Recovered *oriC* plasmid (%),” as means with SD (*n* = 2).

DnaA R281A is impaired in stable DnaA assembly on *oriC*, resulting in largely reduced binding of DnaB compared with wild-type DnaA, although DUE unwinding activity is sustained (Felczak and Kaguni, 2004). Arg281 is a constituent of the AAA+ Box VII motif, which is thought to reside at the interface of DnaA oligomers, supporting stable DnaA–DnaA interactions. To assess the activities of DnaA H136A in stable construction of DnaA assembly on *oriC*, we performed a pull-down assay using biotin-tagged *oriC* fragments. DnaA H136A was recovered by *oriC* fragment pull-down at a similar level to wild-type DnaA (**Figure 6A**), indicating that DnaA H136A is competent for DnaA assembly, which is consistent with its activities in DUE unwinding and ssDUE binding. In the presence of DnaA H136A and DnaB, *oriC* pull-down of DnaB was similar to that in the presence of wild-type DnaA and DnaB, indicating stable binding of DnaB helicase by DnaA H136A (**Figure 6B**). Also, even when DnaC was coincubated, DnaA H136A stably bound DnaB at a level similar to wild-type DnaA (**Figure 6C**). As previously shown (Keyamura et al., 2007), recovery of DnaB was increased by the co-incubation of DnaC, suggesting a conformational change of DnaB by binding of DnaC. Recovery of DnaC was moderately less than that of DnaB, which might be caused by moderate dissociation of DnaC during the wash step in this pull-down experiment.

Loading of DnaB was further examined by a novel pull-down assay using His-tagged DnaB, DnaC, DnaA, IHF, and *oriC* plasmid. In this assay, *oriC* is unwound by DnaA complexes, DnaB undergoes DnaC-dependent loading on the ssDUE region, and the resultant DnaB-bound *oriC* plasmids are recovered with Co^2+^-conjugated beads. The use of wild-type DnaA and *oriC* in this assay resulted in a high level of recovery of *oriC* plasmid, which was dependent on the inclusion of DnaB and DnaC (**Figure 6D**), suggesting that stable complexes with DnaB hexameric rings (or spirals) encircling the ssDNA of *oriC* are necessary for *oriC* recovery. A low level of recovery of *oriC* plasmid in the absence of DnaC presumably represented direct binding of DnaB to DnaA oligomers on *oriC* involving DnaA Phe46. These complexes were likely to have been largely dissociated by the use of wash buffer. In agreement with this idea, the use of wild-type DnaA and mutant *oriC* with DUE deletion (ΔDUE), which is inactive for unwinding, only resulted in a low level of recovery of *oriC* plasmid. Moreover, the use of DnaA F46A resulted in no observable recovery of *oriC* plasmid (**Figure 6D**). These results were consistent with DnaA Phe46-dependent binding between DnaB and DnaA oligomers constructed on *oriC* being responsible for the basal recovery level, with considerable enhancement of recovery resulting from DnaB loading on ssDNA.

The inclusion of DnaA H136A and wild-type *oriC* in this assay in the presence of DnaB and DnaC resulted in moderate inhibition in *oriC* recovery, compared to the level seen with wild-type DnaA (**Figure 6D**). A low level of recovery (similar to that with wild-type DnaA) was seen with DnaA H136A in the absence of DnaC or in the presence of ΔDUE. These results further support the idea that DnaA H136 residue is specifically important for functional DnaB loading to the unwound site of *oriC* (see also section “Discussion”).

### DnaA H136A Is Active in DnaB Loading in a Simplified System

**FIGURE 7:**
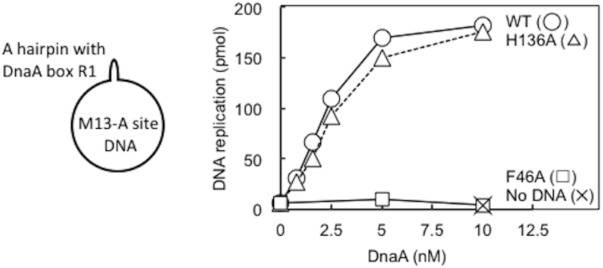
ABC primosome assay. The indicated concentrations of wild-type (WT) or F46A or H136A mutant DnaA were incubated for 15 min at 30°C in buffer containing DnaB, DnaC, DnaG, SSB, NTPs, dNTPs, and DNA polymerase III holoenzyme, with or without M13 A-site ssDNA (220 pmol nucleotides). DNA replication was quantified by measurement of incorporation of radiolabeled dATP. Similar results were obtained when reactions were incubated at 37 or 42°C.

DnaA–DnaB interactions were also studied using the simplified reconstituted ABC primosome system, which uses M13-phage-derived ssDNA with a hairpin structure containing the DnaA box R1 sequence (A-site ssDNA) (Masai et al., 1990; Masai and Arai, 1995; Carr and Kaguni, 2002; **Figure 7**). DnaA binding to the hairpin structure enables recruitment of DnaBC complex and DnaB loading to the SSB-coated ssDNA, followed by primer and DNA synthesis by DnaG primase and DNA polymerase III holoenzyme. Whereas DnaA F46A was essentially inactive in this system, DnaA H136A was fully active relative to wild-type DnaA (**Figure 7**), indicating that DnaA H136A promoted DnaB loading to ssDNA in this simple system. Notably, unlike the more complex *oriC*, only 2–4 DnaA molecules bind to this M13 hairpin region, with its single DnaA box (Carr and Kaguni, 2002), and except for the hairpin region, the whole template is single-stranded. These specific features may cause the different requirement for His136 between *oriC* and A-site ssDNA (see section “Discussion”).

### Evolutional Conservation of DnaA His136

**FIGURE 8:**
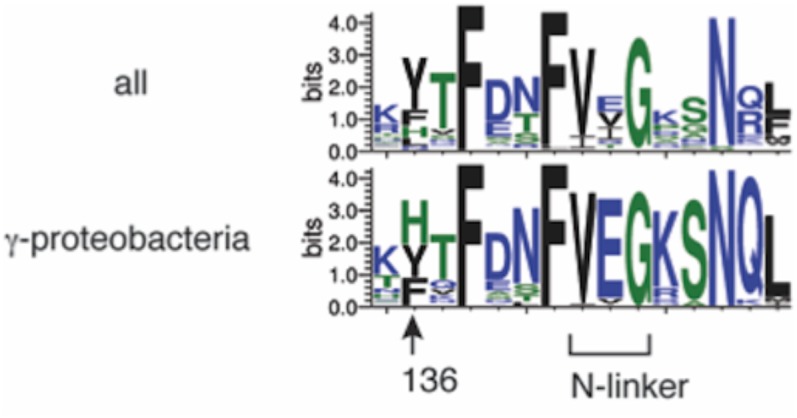
Bacterial DnaA orthologs possess a conserved aromatic residue at the position corresponding to DnaA His136. Sequence logos of DnaA His136 and its surrounding residues are shown based on the sequence alignment of 581 DnaA homologs from all bacterial phyla (top) and 162 DnaA homologs from γ proteobacteria (bottom). The protein sequences were obtained from the NCBI database and the logos were created using WebLogo (http://weblogo.threeplusone.com).

Among bacterial DnaA orthologs, the position corresponding to *E. coli* DnaA His136 is generally occupied by an aromatic residue, such as tyrosine, phenylalanine, or histidine (**Figure 8**). Even in the hyperthermophile *A. aeolicus* DnaA ortholog, the corresponding residue is tyrosine (Erzberger et al., 2002). In γ proteobacteria including *E. coli*, the histidine residue predominates (**Figure 8**). Thus, the aromatic moiety at the position of *E. coli* DnaA His136 appears to have an evolutionarily conserved role in the loading of DnaB helicases at *oriC*.

## Discussion

Loading of replicative helicases on unwound origin DNA region is a crucial step in replication initiation of chromosomes. In *E. coli*, this step depends on dynamic interactions between the initiator DnaA and DnaB replicative helicase. Stable binding between the two depends on a site including DnaA Glu21 and Phe46 of domain I. This site is suggested to interact with a specific site of DnaB, which resides in a region including the N-terminus and its franking region of DnaB CTD. In addition, the DnaA region Lys135–Gln148 within domain III has been implicated as having weak physical contact with DnaB NTD. However, the biological importance of this interaction has not previously been determined. Here, we used alanine scanning of these DnaA residues to highlight the role of His136 in replication initiation *in vivo*. The plasmid complementation test and flow cytometry analysis demonstrated that the *dnaA H136A* allele has only low *in vivo* initiation activity. In-depth biochemical characterization of DnaA H136A revealed that His136 is indispensable for DnaB loading at *oriC*, but that DUE unwinding, ssDUE binding, and assembly on *oriC* do not depend on this residue. Intriguingly, DnaA H136A was fully active in an *oriC*-independent DnaB loading system for ssDNA replication (i.e., ABC primosome system). These observations suggest that DnaA His136 residue has a distinct role in DnaB loading at *oriC*. Because DnaA H136A-*oriC* complexes maintain the primary contact between DnaA Phe46 and DnaB, we conclude that a secondary, weak contact via His136 might allow DnaA-directed DnaB loading on the ssDUE region. The conservation of His136 suggests that its role is conserved in eubacterial DnaA orthologs.

**FIGURE 9:**
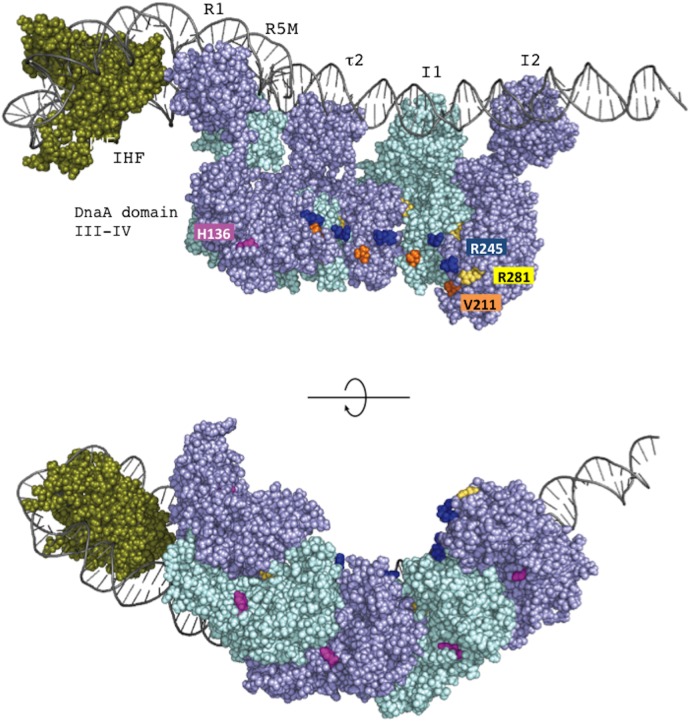
The position of DnaA H136 residue in the left-half DnaA subcomplex. On the basis of a molecular-dynamics-simulation near-atomic model of the *Escherichia coli* initiation complex (Shimizu et al., 2016), the left-half *oriC* complex and important functional residues are shown. In this model, ATP-DnaA domain III–IV molecules are shown complexed with five DnaA boxes (R1, R5M, τ2, I1, and I2) in the left-half DnaA-oligomerization region (DOR). Also, a DNA-bending protein integration host factor (IHF) binds to a unique IHF-binding site. The left-half *oriC* DNA is shown as silver lines. DnaA domain III and IV molecules are shown in violet and light blue. His136 residues are colored pink, and Arg281 residues are colored yellow. ssDUE-binding H/B-motif residues Val211 and Arg245 are shown in orange and dark blue, respectively. IHF is shown in dark green. Two sides of the complexes are shown (upper and lower panels).

Loading of DnaB at *oriC* relies on stable formation of DnaA oligomers on *oriC*. DnaA Arg281 residue indirectly facilitates DnaB binding and loading by contributing to stable binding of DnaA molecules to *oriC* (Felczak and Kaguni, 2004). By contrast, the results of the pull-down assay using an *oriC* fragment indicated that the numbers of DnaA and DnaB molecules bound to *oriC* were similar irrespective of whether wild-type DnaA or DnaA H136A was used. Thus, unlike DnaA K281A, DnaA H136A constructs stable complexes at *oriC* which are fully competent in stable DnaB binding. In our highly detailed structural model of DnaA (Shimizu et al., 2016), the location of the His136 residue suggests that it is unlikely to be involved in interactions between DnaA protomers, unlike the Arg281 residue, which resides at the inter-protomer interface (**Figure 9**).

Our previous *in vitro* and *in vivo* studies as well as the present data are in support of the ssDUE-recruitment mechanism in which DnaA oligomers constructed on DOR stably bind ssDUE using DnaA domain III H/B-motifs (**Figure 1B**; Ozaki and Katayama, 2012; Sakiyama et al., 2017). Recent structural analysis also supports this mechanism (Shimizu et al., 2016). In the paper of Duderstadt et al. (2010), DnaA oligomer formation was moderately stimulated by 25-mer ssDNA and largely decreased by the addition of 13-mer dsDNA bearing a single R1 box, resulting in an increase of DnaA monomers. It should be noted in these experiments that glutaraldehyde cross-linking was used because of the instability of ssDNA binding to DnaA and that this cross-linking produced considerable amounts of DnaA oligomers (dimers to tetramers) even in the absence of ssDNA, causing high background levels. Those results mean that addition of 13-mer dsDNA bearing the R1 box inhibits oligomerization of DnaA and the resultant R1-bound DnaA monomers (but not oligomers) do not bind stably to the ssDNA (and do not mean that dsDNA binding and ssDNA binding of DnaA mutually exclusive). This is consistent with our previous data. We demonstrated that unlike DnaA oligomers constructed on DOR fragments bearing multiple DnaA-binding sites, DnaA bound to a short DNA bearing only a single R1 box is inactive for stable ssDUE binding (Ozaki and Katayama, 2012). In addition, we demonstrated that two T-rich regions in the ssDUE are essential for the binding of ssDUE to DnaA oligomers constructed on the DOR (Ozaki et al., 2008). Consistently, we recently showed that two DnaA molecules bound to the R1 and R5M boxes are crucial for ssDUE binding (Sakiyama et al., 2017). As previously explained (Katayama et al., 2017; Sakiyama et al., 2017), these results concordantly support our idea that when DnaA molecules form an oligomer on the left-half DOR, stable ssDUE binding occur as a result of the linkage effect that enhances cooperative binding (Stauffer and Chazin, 2004; **Figure 1B**).

Our present results highlight a functional relationship between DnaA His136 and Phe46 (**Figure 5**). We previously reported that the DnaA domain I Phe46 exhibits high-affinity binding to DnaB when DnaA oligomers are constructed on *oriC* (Keyamura et al., 2009). In addition, although DnaA F46A alone has little or no activity for DnaB loading, DnaA F46A contributes to DnaB loading in the presence of wild-type DnaA. Similarly, we have now found that DnaA H136A alone has little or no activity for DnaB loading, but it participates in helicase loading at *oriC* when mixed with wild-type DnaA. This result suggests that His136 and Phe46 are only required in a subset of protomers in DnaA oligomers constructed on *oriC*. Further studies are required to determine which protomers in the DnaA subcomplexes might functionally interact with DnaB during the loading processes.

In the *oriC* plasmid-pull down assay using His-DnaB (**Figure 6D**), inhibition by DnaA H136A was moderate compared to the severe inhibition of Form I^∗^ production (**Figure 4A**). Given that DnaA Phe46-dependent DnaB interaction is intact even in DnaA H136A, a possible explanation to this is that interaction of DnaB with DnaA H136A at *oriC* results in abortive loading complexes of DnaB that are not competent for helicase activity. Alternatively, loading orientation of DnaB helicases on ssDUE or DUE strand-specific loading of DnaB might be compromised by the DnaA H136A mutation, resulting in abortive complexes.

Two distinct DnaB-binding modes of DnaA seem to be required for the loading of DnaB onto ssDUE. Notably, heterologous complexes formed at *oriC* by a combination of DnaA domain I F46A and domain III H136A are substantially inactive in DnaB loading (**Figure 5B**), suggesting that both Phe46 and His136 must be present in identical DnaA protomers for helicase loading at *oriC*. Although it has previously been suggested that a weak contact between DnaB and a DnaA region consisting of residues 111–148 (which includes His136) may precede stable binding involving DnaA domain I (Sutton et al., 1998), our results demonstrated that DnaA H136A fully sustained DnaA domain I-dependent DnaB binding. In a previous paper (Sutton et al., 1998) in which Surface Plasmon Resonance analysis was employed, the immobilization of DnaB on the sensor tip may have spatially occluded the DnaA domain I-binding site of DnaB, preventing DnaB interaction with DnaA. Taken together, a likely process is that DnaB binds to DnaA–*oriC* complexes via a primary contact mediated by DnaA domain I, followed by a secondary, weak contact mediated by DnaA His136. Binding of DnaB to DnaA domain I might bring DnaB into proximity with DnaA domain III, enabling the secondary weak contact through DnaA His136 of the same protomer (**Figure 1B**).

**FIGURE 10:**
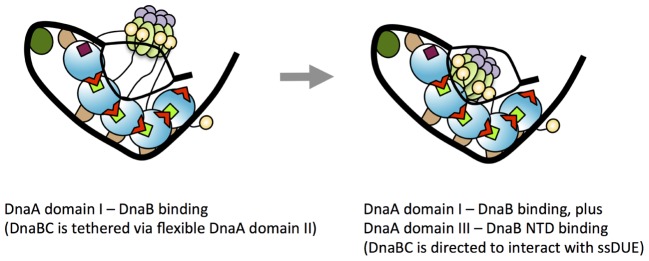
A model for dynamic DnaA–DnaB interactions toward DnaB loading to ssDUE. Possible processes of DnaB loading to the unwound DUE are shown. In the unwound complex, T-rich stand of ssDUE is bound to DnaA subcomplex constructed on the right-half DOR. At the first stage of DnaB loading, DnaB helicase stably binds to multiple molecules of DnaA bound to the DOR as shown in the left panel of this figure (for simplicity, the left-half *oriC*–DnaA subcomplex is shown here). At this point, DnaB in DnaBC complex binds to DnaA domain I and DnaBC is tethered via the flexible DnaA domain II loop, allowing for dynamic motion of DnaBC. In the next step (the right panel), DnaB specifically interacts with domain III His136 of the DOR-bound DnaA, which regulates the position and orientation of DnaBC, facilitating interaction with ssDUE (A-rich strand) and loading of DnaB. For DnaBC tethered to the right-half DOR–DnaA subcomplex, similar changes depending on DnaA His136 occur for DnaB loading to the expanded ssDUE (for details, see Ozaki and Katayama, 2012; Shimizu et al., 2016). Interaction between DnaA domain III and DnaB NTD as well as that between DnaA domain I and the DnaB CTD N-terminus-flanking region is suggested (Seitz et al., 2000). DnaC is suggested to bind to DnaB CDT (Galletto et al., 2003).

We now suggest a model in which the secondary, weak contact via His136 facilitates accessibility of DnaB to the ssDUE (**Figure 10**). Because the T-rich strand of the ssDUE directly binds to the H/B motifs of DnaA domain III (Ozaki et al., 2008; Duderstadt et al., 2011), DnaB bound to DnaA domain I can be brought in close proximity to the ssDUE through the physical contact with DnaA His136 and structural change of the flexible linker domain II (**Figure 10**). Notably, in our high-definition structural model of DnaA–*oriC* complexes, DnaA His136 residues are exposed on the surface of the DnaA protomers, suggesting that they are accessible to DnaB without physically obstructing ssDUE binding to the H/B-motifs (Shimizu et al., 2016; **Figure 9**).

A role for DnaA His136 in regulation of loading of DnaB helicases to the ssDUE might explain why this residue is dispensable for DnaB loading in the ABC primosome system. The ssDNA template in the ABC primosome system most likely minimizes spatial constraints for DnaB loading, thereby bypassing the strict requirement for H136-mediated positioning of the helicase. In other words, ssDNA replication by the ABC primosome system does not require unwinding of dsDNA, regulated DnaA assembly like that on the left-half DOR–IHF complex or loading of two DnaB helicases in opposite directions. By contrast, at *oriC*, DnaB loading must occur on a short singled-stranded region of the DUE. At the M13-A site, the ssDNA region is much larger and thus DnaB loading is less spatially restricted; i.e., DnaB-loading may not be strictly regulated in the ABC promosome system; this may explain the differences in the roles of His136 at *oriC* and in the ABC promosome. Alternatively, SSB that coats the template ssDNA might have an auxiliary role in directing DnaB helicase for ssDNA loading in the ABC primosome system. Functional interaction between SSB and DnaB is reported to stimulate DnaB helicase activity (Biswas et al., 2002).

The linkage effect means that the presence of multiple contact points can result in a drastic increase in binding avidity (total affinity) even if each individual contact only has weak affinity (Stauffer and Chazin, 2004). A similar mechanism might underlie the DnaB interaction that depends on DnaA H136 residues in the *oriC* system. DnaA H136 residues are predicted to be regularly arrayed on the *oriC*-bound DnaA oligomers (**Figure 9**), which might enable formation of multiple contact points with a single DnaB homohexamer. By contrast, in the ABC primosome system, only a few DnaA protomers are involved in a region bearing only a single DnaA box (Masai et al., 1990; Carr and Kaguni, 2002), suggesting that the specific DnaA oligomer structure causing the His136-dependent linkage effect would not be constructed.

Whereas DnaA His136 was dispensable in the ABC primosome system, DnaA domain III is important. Specifically, two residues (Lys234 and Arg285) exposed on the protein surface that are important for DnaA–DnaA interaction stimulate ssDNA replication in the ABC primosome system (Kawakami et al., 2005; Ozaki et al., 2008). Binding of a few DnaA molecules to the hairpin structure of the template ssDNA (Carr and Kaguni, 2002) might be stimulated by domain III-dependent DnaA–DnaA interactions.

Dynamic protein complexes are constructed and change structurally at *oriC* for duplex unwinding and helicase loading. This study reveals the essential function for DnaA domain III His136 in the loading of DnaB replicative helicases on the ssDUE. Further analyses are required to dissect the DnaA protomers responsible for the DnaB interaction during DnaB loading on ssDUE and the DnaA-interacting sites on DnaB as well as to uncover the dynamic mechanisms of DnaA–DnaB complexes underlying the loading orientation of DnaB on ssDUE and DUE strand-specific loading of DnaB.

## Author Contributions

YS, MN, SO, and TK conceived the experiments and wrote the paper. YS, MN, YA, and CH performed the experiments. All authors analyzed the data.

## Conflict of Interest Statement

The authors declare that the research was conducted in the absence of any commercial or financial relationships that could be construed as a potential conflict of interest.
